# Subject-specific stability of toothbrushing performance: insights from dynamic time warping and phenotype analysis

**DOI:** 10.1007/s00784-026-06997-3

**Published:** 2026-07-16

**Authors:** Katja Jung, Carolina Ganss, Elisa-Maria Ressel

**Affiliations:** 1 University Dental Medicine, Clinic for Operative Dentistry, Endodontics, and Pediatric Dentistry, Section for Cariology, Marburg University, Marburg, Germany; 2 Department of Cariology, Periodontology, and Endodontology, Dental Clinic, Justus Liebig University, Giessen, Germany

**Keywords:** Longitudinal study, Habitual toothbrushing, Preventive dentistry, Video-observation, Video instructions, INTERACT

## Abstract

**Objectives:**

This study investigated whether habitual toothbrushing performance is stable within individuals across repeated observations, whether brushing performance changes after different experimental conditions and whether chewing frequency is associated with rhythmic brushing variables.

**Materials and methods:**

In this randomized crossover study, 65 healthy young adults (24.6 ± 2.7 years) completed two study visits. At each visit, habitual manual toothbrushing was video-recorded before and after the assigned experimental condition, which consisted of brushing either after gum chewing or landscape-video viewing. Brushing performance was analysed with regard to active brushing time, surface-specific time allocation, switching behaviour, and brushing stroke rate. Sequence similarity was quantified using dynamic time warping (DTW), and brushing phenotypes were identified by k-means clustering of baseline spatiotemporal features.

**Results:**

Time spent on oral, vestibular, and occlusal surfaces did not differ significantly after chewing (all *p* ≥ 0.300) or landscape-video viewing (all *p* ≥ 0.340). Baseline within-subject DTW distances were markedly lower than between-subject distances (0.38 ± 0.16 vs. 0.69 ± 0.12), corresponding to a 93.4% probability that a randomly selected within-subject distance was smaller than a randomly selected between-subject distance. Three brushing phenotypes (clusters) were identified: (I) long-dwell/systematic (*n* = 13); (II) local-switching (*n* = 23); and (III) global-hopping (*n* = 29). All core spatiotemporal features differed significantly across clusters (all *p* < 0.001). Brushing stroke rate showed very high cross-session consistency (Spearman’s ρ = 0.90–0.95) and a weak positive association with chewing frequency (ρ = 0.28–0.34), whereas switching rate showed no such association.

**Conclusions:**

Core features of toothbrushing performance appear highly stable within individuals and are not meaningfully altered by brief contextual manipulations. Toothbrushing performance therefore reflects habitual execution, but in distinct subject-specific phenotypes.

**Clinical relevance:**

Stable but heterogeneous brushing routines may help explain why oral hygiene often remains suboptimal despite toothbrushing instructions and hands-on-trainings.

**Supplementary Information:**

The online version contains supplementary material available at 10.1007/s00784-026-06997-3.

## Introduction

Toothbrushing is among the most widespread preventive health behaviours and is commonly performed with high regularity in daily life. Yet, observational evidence suggests that the quality of habitual toothbrushing often remains insufficient [1, 2], with characteristic patterns such as uneven time allocation across tooth surfaces, frequent switching between areas, and neglect of oral (i.e. palatal/lingual) surfaces [3–6]. This discrepancy between high behavioural prevalence and limited effectiveness highlights that toothbrushing is not merely a matter of knowledge or motivation, but also of how the behaviour is executed in everyday routines.

A useful behavioural framework to explain these persistent execution patterns is habit. Habits are learned cue–response associations that become increasingly cue-triggered and automatic with repetition in stable contexts [7]. For complex health behaviours, automaticity is not monolithic. Contemporary models distinguish habitual instigation (automatic initiation of the behaviour) from habitual execution (automation of the behavioural sequence once started) [8, 9]. This distinction is directly relevant to oral hygiene. Individuals may reliably start brushing because it is embedded in stable morning/evening routines, while the movement pattern (e.g., sequence, coverage, pacing) remains suboptimal or resistant to deliberate change.

Dental-specific evidence supports this interpretation. Patient narratives indicate that brushing is frequently embedded in broader cleansing scripts and initiated by salient cues (e.g., bathroom-related triggers and event sequences), consistent with cue-dependent habitual initiation [10]. Conceptual work has further described toothbrushing as a routinized “script” that can run with little conscious monitoring once initiated, which may help explain the stability of brushing habits and the difficulty of sustaining changes in everyday contexts [11].

If execution patterns are indeed highly routinized, their stability may extend beyond the order in which tooth surfaces are brushed or the overall coverage achieved. It may also be reflected in the temporal organisation of the behaviour itself, including how quickly and rhythmically brushing movements are produced. From this perspective, rhythm is not a separate phenomenon, but a potentially integral feature of habitual execution.

Accordingly, motor rhythm represents a relevant additional dimension of toothbrushing behaviour. Kinematic analyses indicate that toothbrushing can be described as cyclic motor behaviour with measurable frequency and individual-specific rhythmic profiles linked to arm–joint coordination [12]. A plausible neurophysiological counterpart for rhythmic stability is chewing, a prototypical rhythmic orofacial behaviour generated by brainstem circuitry (the masticatory central pattern generator) and modulated by sensory feedback [13]. These considerations lead to the question of whether rhythmic parameters of toothbrushing (e.g., brushing stroke) remain stable within individuals over time and whether they correlate with chewing frequency as another rhythmic motor measure.

At the same time, execution may be sensitive to state manipulations that differ in attentional engagement. Mastication has been associated with small improvements in sustained attention in healthy adults, and chewing gum has been discussed in relation to alertness and cognitive performance [14, 15]. In contrast, passive exposure to nature/landscape videos has been used to induce relaxation or stress reduction in experimental paradigms [16, 17]. Comparing an active rhythmic manipulation (gum chewing) with a passive visual (landscape video) condition therefore provides a pragmatic way to test whether changes in brushing execution are more consistent with motor/rhythm-related influences versus non-motor, affective context changes.

Against this background, the present study used repeated, standardised observational assessments of habitual brushing to characterise (i) the stability of individual execution patterns across timepoints, (ii) whether brushing execution differs depending on experimental conditions, and (iii) whether chewing frequency covaries with brushing-related rhythmic parameters, particularly brushing stroke rate and switching rate.

### Participants, materials and methods

This study was a randomized crossover study with two different experimental conditions and video-based observational analyses. The study was conducted at the Department of Conservative and Preventive Dentistry, Dental Clinic, Justus-Liebig -University Giessen, Germany, in accordance with Good Clinical Practice (GCP) and the Declaration of Helsinki (2013). Ethical approval was obtained from the local Ethics Committee (Doc. No. 186/20), and the trial was registered in the German Clinical Trials Register (DRKS00033844).

Participants were eligible if they were 18 years or older, provided written informed consent, and had no professional background in medicine or dentistry. Exclusion criteria comprised severe systemic disease; mental or physical impairment that could compromise oral hygiene performance; known intolerance to chewing-gum ingredients or to study materials; multiple gingival recessions extending beyond one third of the root length; defective restorations; cavitated carious lesions; fewer than 24 permanent teeth present (excluding third molars); removable dentures, fixed bridges, or gap dentition; fixed orthodontic appliances (retainers allowed); and dental malformations.

### Study procedure

Participants were recruited via posters at central locations at Justus-Liebig-University Giessen and via the notice board. Interested individuals contacted the investigator (E.R.), and their suitability was checked in advance by telephone or e-mail using a standardised checklist for inclusion and exclusion criteria.

**Fig. 1:**
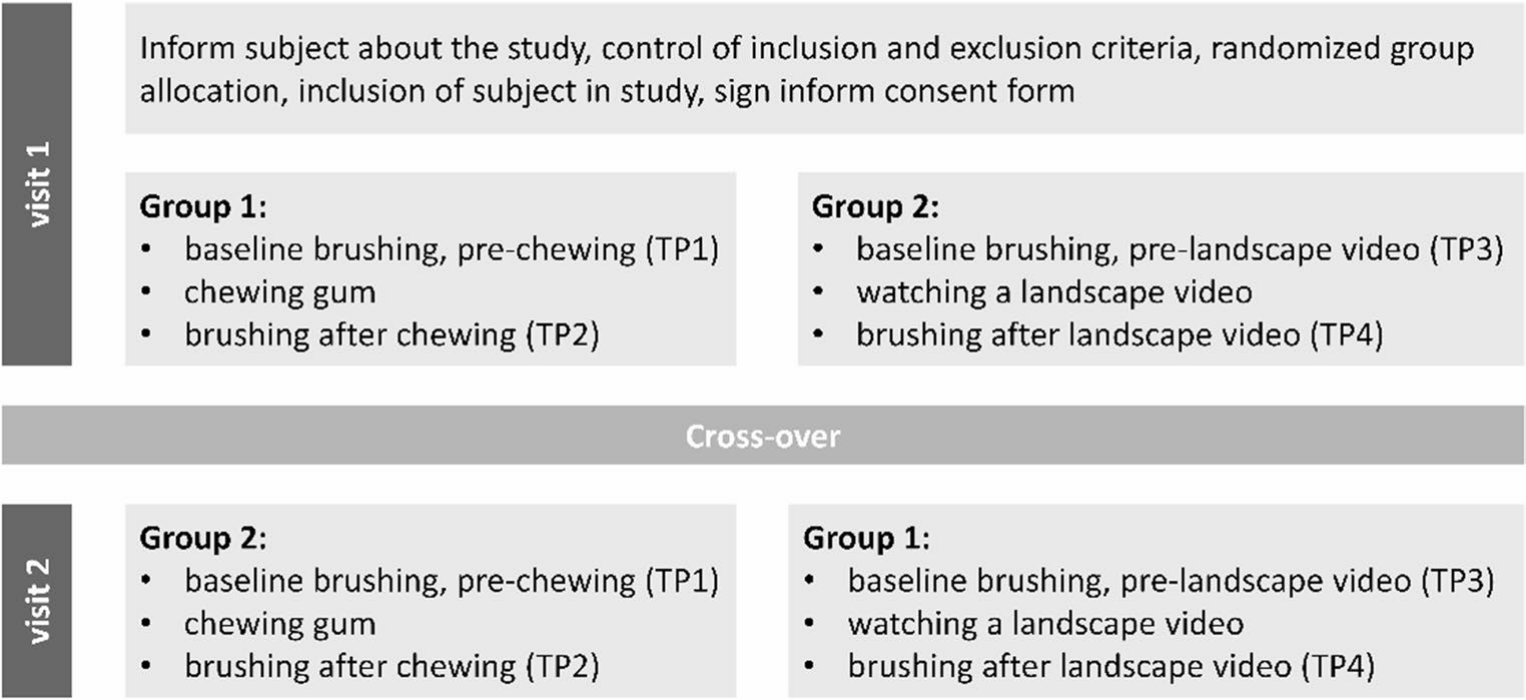
Flow chart

After eligibility screening and provision of written informed consent at the first appointment, a total of 65 participants (mean age 24.6 ± 2.7 years) were included. Participants were assigned according to a randomization list to one of two experimental conditions (Group 1 vs. Group 2) in a cross-over design (Fig. 1). Each participant received a pseudonymised study code used for file naming and data management.

At each study visit (visit 1 and visit 2), participants performed habitual toothbrushing using a standardized manual toothbrush (elmex^®^ CARIES PROTECTION interX, short brush head, medium; CP GABA, Hamburg, Germany). To facilitate subsequent video-based behavioural coding, toothpaste was omitted. Toothbrushing was performed in front of a mirror device with an integrated camera opening; a 4 K Ultra-HD camcorder (Panasonic HC-VX878, Kadoma, Osaka, Japan) recorded the procedure. No study personnel were present in the room during recordings to minimize behavioural reactivity. Video recording was started manually and saved under the participant’s study code.

Following the baseline toothbrushing recording, participants completed one of two experimental conditions: brushing after chewing gum (2 min; Wrigley’s EXTRA Professional Peppermint Mini-Strips, Mars GmbH, McLean, VA, USA) or after watching a standardized landscape video (4 min) with calm background music. At visit 1, Group 1 used chewing-gum and Group 2 watched the landscape video. After a 2-week interval, participants returned for visit 2 and crossed over to the alternate experimental condition. During chewing gum, mandibular movements were recorded frontally, markers were placed at the nasal tip and chin to quantify chewing frequency relative to a fixed reference point.

Immediately after the gum chewing or landscape video, toothbrushing was recorded again.

TP1 = baseline brushing, pre-chewing, TP2 = brushing after chewing, TP3 = baseline brushing, pre-landscape video, TP4 = brushing after landscape video.

Thus, TP2 vs. TP1 represented the chewing contrast, TP4 vs. TP3 the landscape contrast, and TP1 vs. TP3 baseline stability across visits. The study design is illustrated in Fig. 1.

### Video analysis

After all participants had completed both visits, video analysis was performed using INTERACT (version 9.6.1.170, Mangold International, Arnstorf, Germany). The analysis required several passes and was based on parameters described in the doctoral thesis by Tobias Winterfeld [18]:

- **Active brushing time** was defined as the actual time the toothbrush was in contact with the teeth, excluding interruptions such as rinsing, spitting, or breaks.
- **Sextants** were defined by subdividing the maxillary and mandibular arches into sextants (S1–S6). For toothbrushing conducted with the jaws closed, additional paired sextants were defined as S7: S1_6, S8: S2_5, and S9: S3_4.
- **Surface** was coded as vestibular (i.e., buccal/labial), occlusal, or oral (i.e., palatal/lingual), depending on the region of the teeth reached during brushing.
- **Toothbrushing performance** was coded from video as time-stamped intervals (onset/offset in ms) describing the sextant (coded 1–9) and surface (oral/vestibular/occlusal). Because sextant and surface were annotated in separate event streams, a combined spatiotemporal state was derived by intersecting intervals from the sextant stream with intervals from the surface stream, yielding contiguous segments labelled by sextant × surface. Segment durations were computed as offset − onset and were expressed in milliseconds (and converted to seconds where indicated).
- **Switching rate** was defined as the rate of alternations between consecutively brushed areas and is reported as switches per minute of active brushing time.
- **Brushing strokes** were defined as each reversal of brushing movement direction.
- **Brushing stroke rate** was defined as the number of brushing strokes per unit time and calculated as stroke rate = n / t (strokes/s), where n is the total number of direction reversals and t is the corresponding brushing time.
- **Chewing strokes** were defined as each reversal of lower-jaw movement direction in the vertical plane (opening–closing).
- **Chewing frequency** was defined as the number of chewing strokes per unit time and calculated as chewing frequency = n / t (strokes/s), where n is the total number of jaw-direction reversals and t is the recording time.

### Inter- and intra-rater reliability

The video analysis required an extensive training phase. For this purpose, video recordings from a previous observational study [18] were repeatedly analysed by E-M.R., ten videos were then randomly analysed for testing inter-rater reliability. Kappa coefficients were obtained directly using the INTERACT software. Coefficients for inter-rater agreement were 0.84 for sextant classification, 0.79 for classification of the tooth surfaces reached (oral, vestibular, or occlusal), and 1.00 for brushing duration in the different sextants of the dentition. For intra-rater calibration, the E-M.R. analysed 10 randomly selected videos twice: once before the main video evaluation and again after half of all recordings had been completed. The kappa coefficients for the first intra-rater calibration were 0.85 for sextant classification, 0.74 for classification of the tooth surfaces reached, and 1.00 for brushing duration. Corresponding values for the second intra-rater calibration were 0.87, 0.77, and 1.00.

### Statistical analysis

Statistical analyses and figure generation were performed in a local Spyder (version 5.4.5) environment using Python 3.10. Data handling and tabulation were performed using pandas and NumPy, statistical tests using SciPy, mixed-effects models using statsmodels, clustering and validation using scikit-learn, and figures using matplotlib.

#### Time allocation during toothbrushing

To test whether time spent brushing different tooth surfaces changed across experimental conditions, surface-specific brushing time (seconds) was analysed using linear mixed-effects models. For each surface (oral, vestibular, occlusal), brushing time per participant and timepoint (TP1-TP4) was computed as the sum of all surface-coded event durations. Timepoint was entered as a categorical fixed effect and a participant-specific random intercept was included to account for repeated measures. Contrasts tested the chewing effect (TP2 vs. TP1), the landscape effect (TP4 vs. TP3), and baseline stability (TP3 vs. TP1). All tests were two-sided with α = 0.05.

#### Sequence similarity of brushing performance

To quantify similarity of brushing sequences across sessions, each session was converted into 100 normalized time bins spanning the session duration. For each bin, a 27-dimensional soft state vector (9 sextant codes × 3 surfaces) was computed, with each element indicating the proportion of bin time spent in that state. Session pairs were compared using dynamic time warping (DTW) with cosine distance between bin vectors and path-length normalization (lower values indicate greater similarity) following the general approach described by Kopland and Giltay [19]. Within-subject comparisons were baseline stability (TP1 vs. TP3), chewing effect (TP1 vs. TP2), and landscape effect (TP3 vs. TP4).

To contrast intra- and interindividual similarity at baseline, DTW distances were computed for (i) within-subject TP1 vs. TP3 pairs and (ii) between-subject all unique TP1 vs. TP1 pairs. As a descriptive effect size, the common-language probability P (within < between) was calculated, defined as the proportion of comparisons in which a randomly selected within-subject DTW distance was smaller than a randomly selected between-subject distance. Further details on the DTW procedure are provided in Supplement Material.

#### Spatiotemporal brushing phenotypes

Spatiotemporal features were derived from run-length encoded sequences of the intersected sextant × surface segments. To reduce inflation of switching counts due to rapid boundary flicker (e.g., at canine transitions), a debounce smoothing procedure was applied by merging (i) identical labels separated by short gaps (≤ 50 ms) and (ii) runs shorter than 300 ms into neighbouring runs. Active brushing time was defined as the summed duration of intersected segments. From the smoothed sequences, dwell time (median and mean run duration, seconds) and switching rate (switches per minute of active brushing time) were computed.

Large-scale movement across the dentition was quantified using a sextant ring-distance metric (1–6 arranged cyclically; combined codes treated as sets 7 = (1,6), 8 = (2,5), 9 = (3,4) with minimum-set distance). Two jumpiness measures were derived: non-adjacent switch rate (ring distance ≥ 2 per minute of active brushing time) and spatial travel (sum of ring distances per minute of active brushing time).

To derive individual brushing phenotypes, a baseline feature profile was computed per participant as the mean of TP1 and TP3 for dwell, switching rate, jumpiness, spatial travel, and motor tempo (brushing stroke rate, strokes/s). Skewed features were log-transformed (log(1 + x)) and z-standardized. K-means clustering (k = 3; multiple random initializations) was performed on three composite axes: Stability = z(log dwell) − z(log switch rate), Exploration = z(log spatial travel) + z(log non-adjacent switches), and Tempo = z(brushing stroke rate). Cluster robustness was assessed using silhouette coefficients and assignment stability across random starts and bootstrap resampling (cluster confidence = proportion of repeated solutions with identical assignment after label alignment). Cluster summaries are reported as median (bootstrap 95% CI). Cluster differences were tested using Kruskal–Wallis with ε² effect sizes and Holm correction; significant omnibus tests were followed by Holm-adjusted Mann–Whitney U post-hoc tests.

#### Relations among chewing frequency, brushing stroke rate, and switching rate

Descriptive statistics (mean ± SD, median [IQR], range) were computed for chewing frequency, brushing stroke rate, and switching rate across all sessions (TP1–TP4 pooled). To assess motor “anchoring,” consistency of brushing stroke rate across sessions was quantified using pairwise Spearman rank correlations between timepoints (TP1–TP4). Associations among chewing frequency, brushing stroke rate, and switching rate were evaluated using Spearman correlations. All tests were two-sided with α = 0.05.

## Results

### Time allocation during toothbrushing

**Fig. 2:**
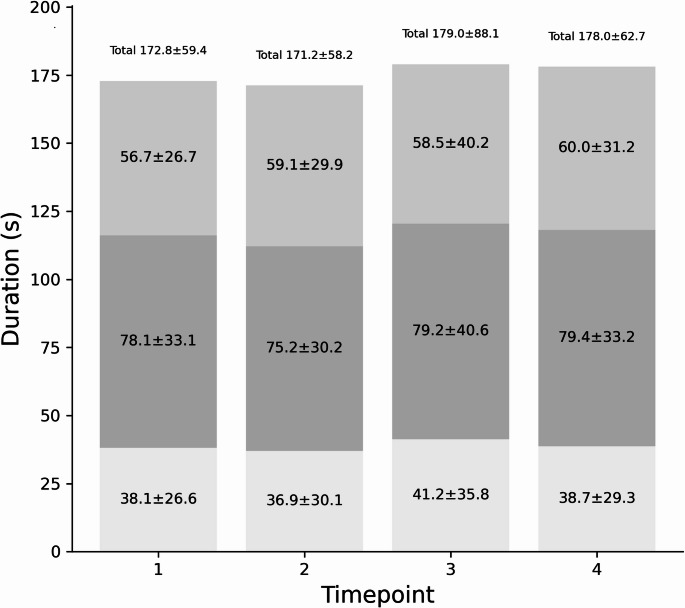
Brushing time across experimental timepoints (TP1 to TP4). Stacked bars show the mean time spent brushing occlusal (medium grey), vestibular (dark grey), and oral surfaces (light grey), values printed within each segment indicate mean ± SD. Values above each bar indicate total active brushing time (sum of oral + vestibular + occlusal durations; mean ± SD). TP1 and TP3 represent baseline (pre) recordings, TP2 brushing after chewing, and TP4 brushing after landscape video

Active brushing time was similar across timepoints (Fig. 2). Linear mixed models (random intercept for participant) showed no evidence that time spent on oral, vestibular, or occlusal surfaces differed across the experimental timepoints. Specifically, the chewing contrast (TP2 vs. TP1) was not significant for oral (*p* = 0.673), vestibular (*p* = 0.395), or occlusal surfaces (*p* = 0.300). Likewise, the landscape contrast (TP4 vs. TP3) was not significant for oral (*p* = 0.340), vestibular (*p* = 0.997), or occlusal (*p* = 0.415). The two baseline sessions also did not differ (TP3 - TP1: oral *p* = 0.268; vestibular *p* = 0.603; occlusal *p* = 0.532).

Consistent with the surface-specific analyses, total active brushing time did not differ between experimental conditions (TP2 vs. TP1: *p* = 0.937; TP4 vs. TP3: *p* = 0.975; TP3 vs. TP1: *p* = 0.324).

### Sequence similarity of brushing performance

**Fig. 3:**
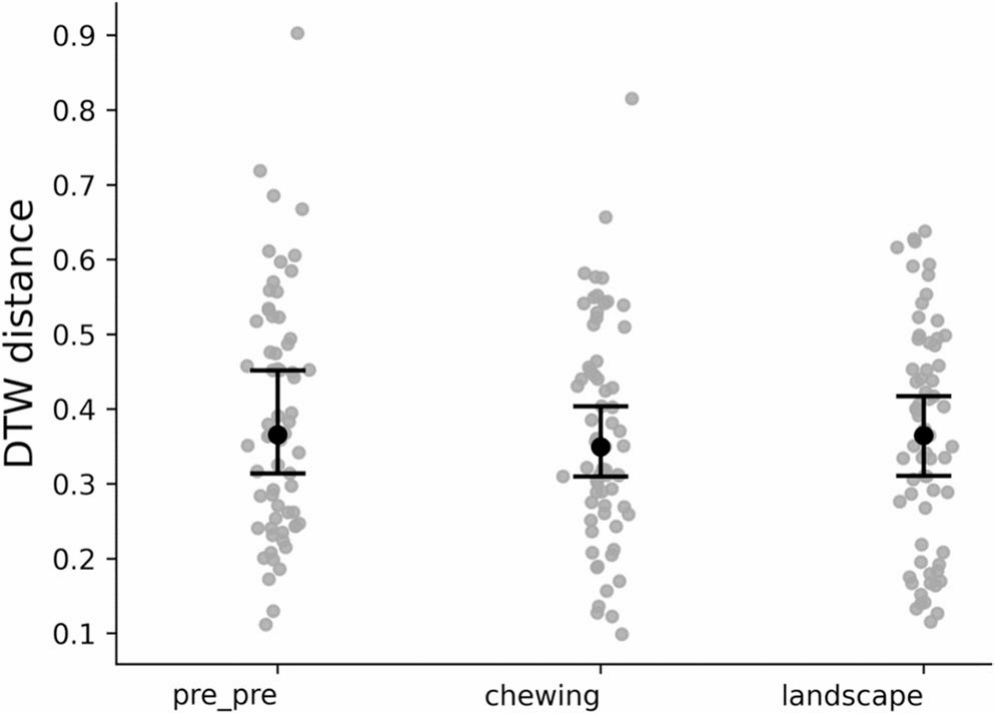
Within-subject similarity of toothbrushing sequences across comparisons. Each grey point represents one participant’s dynamic time warping (DTW) distance between two sessions for the indicated comparison (pre–pre: TP1 vs. TP3; chewing effect: TP1 vs. TP2; landscape effect: TP3 vs. TP4). Black markers indicate the median; whiskers indicate the bootstrap 95% CI of the median (20,000 resamples)

DTW distances were similar across the three prespecified comparisons (Fig. 3; all pairwise p-values 0.235–0.878). Median DTW distances (bootstrap 95% CI) were 0.366 (0.314–0.452) for TP1 vs. TP3, 0.349 (0.310–0.404) for TP1 vs. TP2, and 0.365 (0.311–0.417) for TP3 vs. TP4. Mean ± SD values were comparable (0.390 ± 0.160; 0.366 ± 0.146; 0.363 ± 0.149), while individual DTW distances varied substantially.

**Fig. 4:**
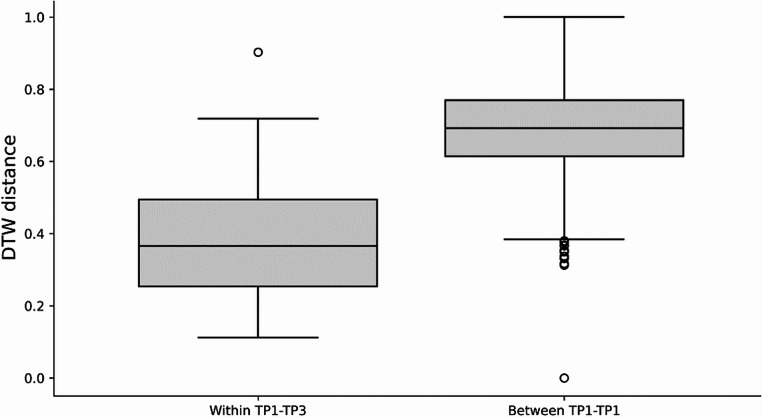
Intra- vs. interindividual similarity of toothbrushing sequences at baseline. Boxplots show the distribution of DTW distances for within-subject baseline stability (TP1 vs. TP3) and between-subject baseline similarity (all unique TP1 vs. TP1 pairs)

Baseline within-subject DTW distances (TP1 vs. TP3; 0.38 ± 0.16) were markedly lower (more similar) than between-subject baseline distances (TP1 vs. TP1; 0.69 ± 0.12), with a 93.4% probability that a randomly selected within-subject distance is smaller than a randomly selected between-subject distance (P(within< between) = 0.934) (Fig. 4), indicating strong individual “fingerprints” of brushing sequences.

### Spatiotemporal brushing phenotypes

Participants clustered into three well-separated brushing types. Cluster separation was moderate-to-good (silhouette score 0.345 in the 3D clustering space; 0.397 in the Stability×Exploration projection), and cluster assignments were highly reproducible (seed-based stability ARI mean 0.823; bootstrap stability ARI median 0.841, 10th–90th percentile 0.610–1.000).

**Fig. 5:**
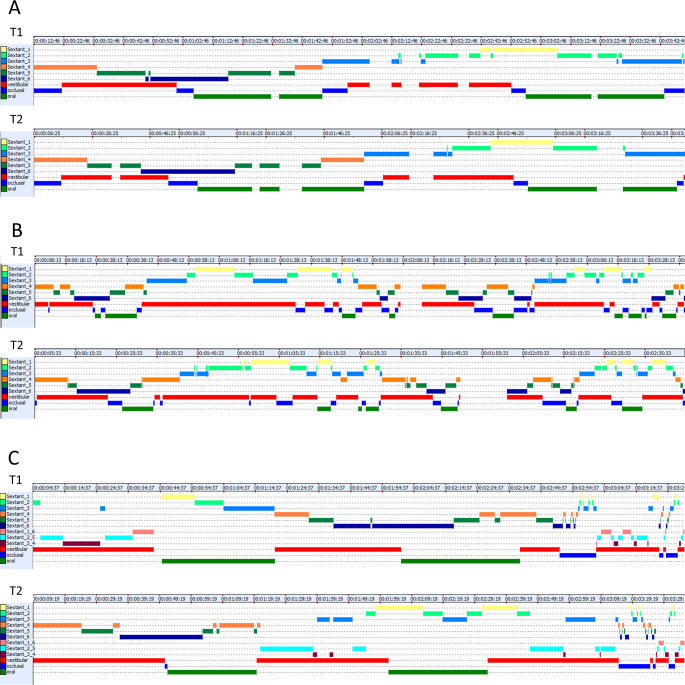
Exemplary time-line charts of brushing behaviour in participants assigned to cluster 1 (A), cluster 2 (B), and cluster 3 (C). Cluster 1 (**A**) reflects a systematic long-dwell phenotype (DTW = 0.130), cluster 2 (**B**) a local switching phenotype (DTW = 0.383), and cluster 3 (**C**) a global hopping phenotype (DTW = 0.903). Panels A–C illustrate representative participants with corresponding positions on the composite dimensions of Stability, Exploration, and standardized brushing tempo.

Cluster 1 (*n* = 13) reflected a systematic/long-dwell brushing phenotype with longer dwell times and lower switching and spatial travel, cluster 2 (*n* = 23) a local switching phenotype with relatively low non-adjacent jumping and cluster 3 (*n* = 29) a global hopping phenotype characterized by short dwell, high switching, high spatial travel, clearly elevated non-adjacent jumps and shorter brushing duration (Table 1; Fig. 5).

Across clusters, differences were significant for all variables (all Holm-adjusted *p* < 0.001) with large effect sizes (Kruskal-Wallis ε² = 0.51–0.76), indicating that phenotype membership accounted for a substantial proportion of variability in the core spatiotemporal features. Active brushing time also differed across clusters, but with a smaller effect size (*p* < 0.001; Kruskal-Wallis ε² = 0.30).

**Table 1:** Cluster characteristics. Values are median [bootstrap 95% CI] based on 20,000 resamples. Different superscript letters denote significant pairwise differences between clusters (Holm-adjusted post-hoc tests)

	Cluster 1 (*n* = 13)	Cluster 2 (*n* = 23)	Cluster 3 (*n* = 29)
Dwell time [s]	3.2 [2.6–3.4] ^a^	1.8 [1.8–2.0] ^b^	1.2 [1.2–1.4] ^c^
Switching rate [switches/min]	9.0 [7.1–10.7] ^a^	19.8 [17.2–22.7] ^b^	30.6 [26.4–34.9] ^c^
Non adjacent switch rate [switches/min]	1.3 [0.5–1.4] ^a^	1.2 [0.9–1.9] ^a^	3.4 [2.6–4.0] ^b^
Spatial travel [ring-distance units/min]	6.4 [5.2–8.5] ^a^	13.5 [11.1–16.6] ^b^	23.0 [21.8–26.7] ^c^
Brushing stroke rate [strokes/s]	7.4 [6.1–8.5] ^a^	8.3 [8.0–8.7] ^ab^	7.7 [7.2–8.0] ^ac^
Active brushing time [s]	201 [183–380] ^a^	203 [174–214] ^a^	126 [113–145] ^b^

**Fig. 6:**
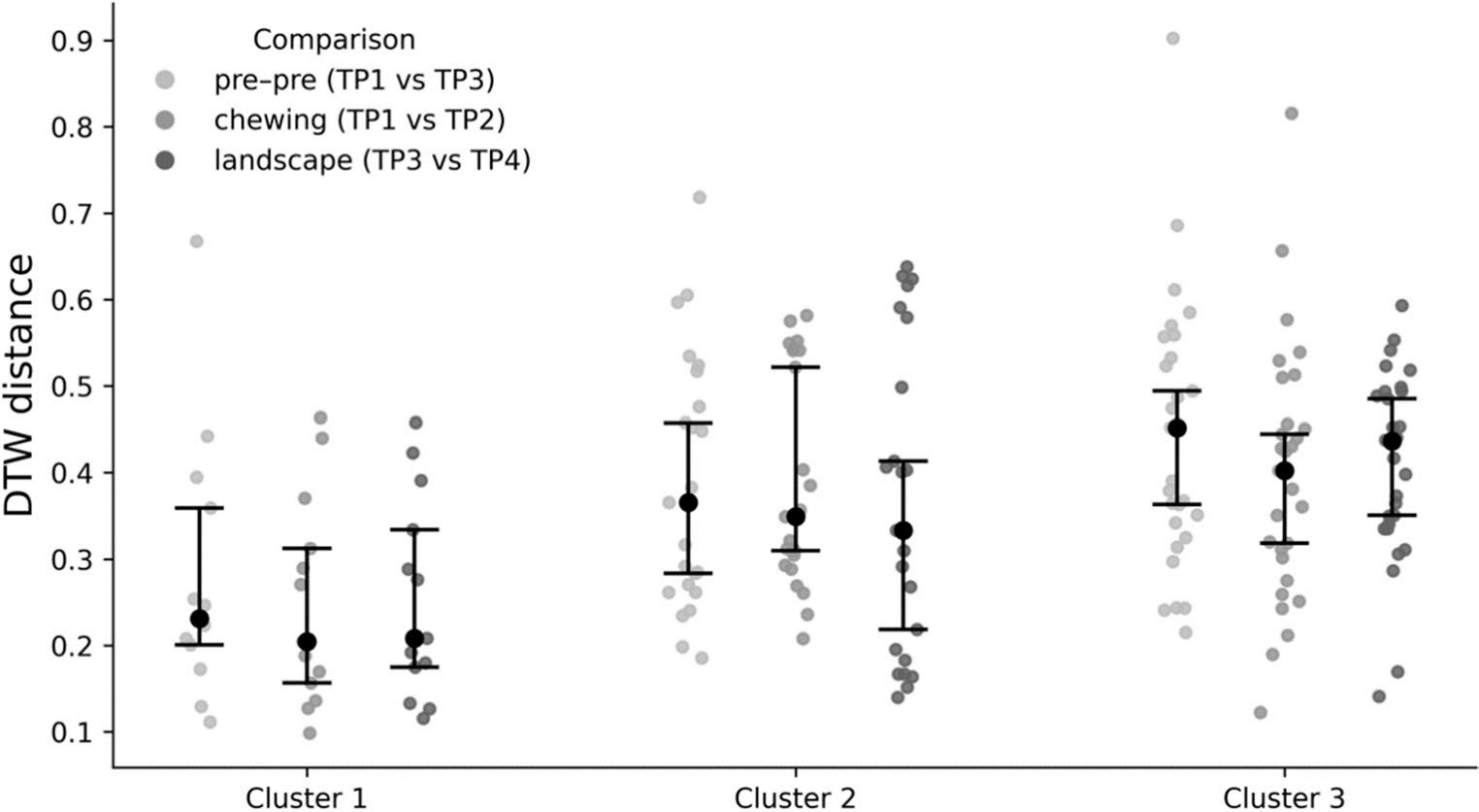
Within-subject similarity of toothbrushing sequences per cluster. Each grey point represents one participant’s dynamic time warping (DTW) distance between two sessions. Black markers indicate the median; whiskers indicate the bootstrap 95% CI of the median (20,000 resamples)

The three brushing phenotypes also differed in sequence similarity of brushing performance for baseline (TP1 vs. TP3; *p* < 0.05) and for the chewing and landscape comparisons (TP1 vs. TP2: *p* < 0.05; TP3 vs. TP4: *p* < 0.05) (Fig. 6). Post-hoc tests indicated that Cluster 1 showed significantly lower DTW distances than Cluster 2 and/or Cluster 3 (TP1 vs. TP3: 1 vs. 2 *p* < 0.05; 1 vs. 3 *p* < 0.01; 2 vs. 3 n.s.; TP1 vs. TP2: 1 vs. 2 *p* < 0.05; 1 vs. 3 *p* < 0.05; 2 vs. 3 n.s.). For landscape (TP3 vs. TP4), the difference was only significant between Cluster 1 and Cluster 3 (*p* < 0.05). A considerable range of values remained within clusters, but even Cluster 3 still had a substantial within-subject similarity.

### Relations among chewing frequency, stroke rate, and switching

Across all sessions (TP1–TP4 pooled; 65 participants × 4 sessions = 260 observations), the median brushing stroke rate was 7.9 s⁻¹ (bootstrap 95% CI 7.2–8.6; mean ± SD 7.8 ± 1.3; range 3.1–10.8), the median switching rate was 21.9 min⁻¹ (95% CI 14.7–30.0; mean ± SD 23.3 ± 12.0; range 4.0–60.2), and the median chewing frequency was 2.8 s⁻¹ (95% CI 2.6–3.0; mean ± SD 2.8 ± 0.4; range 1.8–3.7).

Brushing stroke rate exhibited very high cross-session consistency. Pairwise correlations between timepoints were uniformly strong (Spearman ρ = 0.90–0.95, all p-values < 0.001). For example, brushing stroke rate at TP1 correlated strongly with TP2 (ρ = 0.95), TP3 (ρ = 0.91), and TP4 (ρ = 0.92), and TP3 correlated strongly with TP4 (ρ = 0.95). This indicates that brushing motor tempo is a stable individual characteristic across sessions, even when other aspects of brushing performance differ.

Brushing stroke rate was positively associated with chewing frequency at each timepoint. Spearman correlations were consistently small-to-moderate across TP1 to TP4 (ρ = 0.28–0.34, *p* = 0.02 − 0.006). Thus, individuals who chewed faster tended to show faster brushing strokes.

In contrast, brushing stroke rate and chewing frequency showed no meaningful association with switching rate. Across TP1 to TP4, correlations between chewing frequency and switching rate were small and non-significant (ρ = -0.07 to -0.13, all *p* > 0.29), and brushing stroke rate and switching rate were also essentially unrelated.

## Discussion

The present randomized cross-over study primarily suggests that habitual toothbrushing is characterized by strong within-subject stability at the level of execution. Previous video observation studies have described recurrent, firmly established movement patterns during toothbrushing, including frequent neglect of oral surfaces, repeated returns to certain areas, and recurrent alternation patterns. In the classic work by Rugg-Gunn and Macgregor, adults brushed for much shorter total times (39.1 ± 16.6–79.1 ± 27.4 s) than contemporary cohorts, yet oral surfaces were still markedly underrepresented, whereas vestibular and occlusal areas received substantially more attention [6]. Decades later, Winterfeld et al. [3] reported a mean effective brushing duration of 156.0 ± 71.1 s, but still found that vestibular surfaces were brushed more than twice as long as oral surfaces, and concluded that brushing patterns were highly similar to those observed more than 30 years earlier. Our data fit this pattern closely. Total brushing duration was similar to that reported by Winterfeld et al. [3], and participants in the present study likewise devoted substantially less active brushing time to the oral surfaces than to the vestibular surfaces. Occlusal surfaces still accounted for a considerable proportion of total brushing time, and this distribution remained stable across repeated assessments. Taken together, these findings suggest that although overall toothbrushing duration appears to have increased substantially across generations, the relative allocation of brushing time across dental surfaces may have changed little.

This stability was also evident beyond simple time measures. Brushing duration, surface-specific time allocation, and sequence-related characteristics remained highly reproducible across repeated sessions, and neither chewing nor the landscape-video produced meaningful changes in these parameters. Accordingly, the data do not support the assumption that brief contextual manipulations are sufficient to reorganize habitual brushing performance. Rather, they indicate that core features of brushing performance are relatively resistant to short-term influences and appear to reflect stable execution routines. Moreover, the lack of differential effects after chewing versus landscape video argues against both a simple motor priming account and a non-specific attentional or affective explanation.

These findings should nevertheless be interpreted in light of the exploratory nature and short duration of the experimental conditions. Gum chewing was selected as a brief, standardized orofacial sensorimotor activation. Neuroimaging studies support this rationale by showing that chewing activates motor- and sensorimotor-related brain regions, including the sensorimotor cortex, supplementary motor area, insula, thalamus, and cerebellum [20], as well as the motor cortex, caudate, cingulate cortex, and brainstem [21]. Near-infrared spectroscopy studies further indicate increased oxygenated haemoglobin in the prefrontal cortex during gum chewing, consistent with increased cortical blood flow and activation [22]. Thus, chewing can reasonably be regarded as an active motor-activating condition and has been used previously in cognitive, arousal-related, and motor-learning paradigms, including protocols with 2-min pre-task chewing periods [23, 24].

The landscape-video condition was selected as a passive, low-activation comparator and also served to standardize the interval between the two brushing episodes. Nature and landscape videos have been used as relaxing or restorative stimuli [16], and comparable 4-min neutral or nature-film periods have been used in psychophysiological studies as baseline, acclimatization, or calming phases before affective, stress-related, or cardiovascular assessment tasks [25, 26]. Although the 4-min duration was chosen pragmatically, it is therefore consistent with established experimental practice. Because no independent manipulation checks of attention, alertness, arousal, stress, or relaxation were included, the absence of significant differences after either experimental condition does not demonstrate that chewing or landscape viewing were ineffective at the level of internal state. Rather, it indicates that these brief conditions, as implemented here, were not sufficient to measurably alter habitual brushing execution under the present study conditions. Accordingly, the present findings are consistent with the view that not only brushing instigation, but also key aspects of brushing execution, may be habitualized.

At the same time, this broad cross-generational similarity in surface-specific time allocation should not be mistaken for homogeneous brushing performance. Beneath the stable group-level pattern, participants differed markedly in how they organized brushing over time. This became particularly evident in the sequence-similarity analyses. Dynamic time warping (DTW), a well-established method for comparing temporal sequences that may vary locally in timing or speed [27–29], allowed us to compare entire brushing trajectories rather than isolated summary measures. To the best of the authors’ knowledge, this approach has not yet been applied to observational toothbrushing sequences in dentistry. In the present study, DTW showed that brushing sequences were more similar within than between individuals, indicating that participants tended to reproduce subject-specific execution patterns rather than merely a generic population-level routine.

The phenotype analysis further strengthened this interpretation. Participants could be grouped into three distinct brushing phenotypes, characterized by longer dwell times and greater local stability, more frequent local switching, or more global hopping-like movement through the dentition. These phenotypes differed not only in dwell time, switching, non-adjacent jumping, and spatial travel, but also in the reproducibility of their brushing sequences across repeated sessions. Importantly, reproducibility was not restricted to the more structured phenotype. Although the long-dwell phenotype showed the lowest DTW distances overall, low within-subject DTW distances were also observed in the more irregular phenotypes. Thus, even globally jumpy or seemingly chaotic brushing patterns could be reproduced quite consistently within individuals. At the same time, the greater spread of DTW distances in the local-switching and hopping-like phenotypes indicates that these execution styles were less uniformly stable across participants and allowed for greater within-phenotype variability. Together, these findings indicate that interindividual differences in brushing are not limited to isolated parameters, but reflect broader differences in how brushing performance is organized and stabilized over time. Accordingly, the absence of effects of the experimental conditions at group level should not be interpreted as evidence of uniform brushing behaviour, but rather as stability within a heterogeneous population of subject-specific execution styles.

Overall, the present data therefore argue against the notion of a single habitual brushing style. While the general distribution of brushing time across oral, vestibular, and occlusal surfaces appears strikingly conserved across decades, individuals seem to express distinct brushing types that differ in temporal organization, switching behaviour, spatial exploration, and sequence stability. This is in line with earlier observational work and systematics-based analyses showing that toothbrushing behaviour can be described in structured rather than random terms [3, 30].

Whereas the spatiotemporal organization of brushing remained largely stable, the rhythmic variables showed a more differentiated pattern. Across all sessions, brushing stroke rate showed very high cross-session consistency, indicating that brushing stroke rate is a stable individual characteristic. Brushing stroke rate was also positively associated with chewing frequency, whereas no meaningful association was found with switching rate. This selective covariation suggests a dissociation between motor tempo and spatiotemporal sequence organization. In other words, oral motor tempo may reflect a relatively stable individual rhythmic tendency that generalizes across different orofacial behaviours, whereas switching behaviour appears to capture a different aspect of brushing control that is more closely related to sequencing, coverage organisation, or behavioural strategy than to rhythmic tempo itself.

Habitual chewing frequencies reported in the literature are usually based on complete chewing cycles per unit time. Po et al. reported a mean chewing frequency of 1.57 Hz [31], and Sánchez-Ayala et al. categorized habitual chewing rates into bands below 70, 70–90, and above 90 cycles per minute, (corresponding to < 1.17, 1.17–1.50, and > 1.50 Hz) [32]. By contrast, the chewing variable in the present study was derived from coded vertical jaw movements and is therefore not directly comparable to cycle-based measures. Because opening and closing movements were counted separately, the resulting values would be expected to be approximately twice as high as frequencies based on complete chewing cycles. Accordingly, the absolute chewing-frequency values observed in the present study (2.8 Hz) are broadly compatible with those reported in the literature once this difference in operationalization is taken into account.

This interpretation is compatible with the broader idea that many everyday actions have an oscillatory structure and are shaped by neural circuits involved in rhythmic motor control. In a simultaneous dual-task paradigm, Samulski et al. [33] showed that experimentally altered chewing rates were accompanied by corresponding changes in stepping rate, suggesting that rhythmic motor behaviours can covary across domains under specific task intervention. However, their study does not demonstrate a shared rhythm generator directly; rather, it supports the more cautious view that chewing-related neural drive may influence motor output beyond the orofacial system when behaviours are performed concurrently.

A possible biological substrate for such covariation lies in the orofacial sensorimotor system. Mastication is generated by brainstem circuitry and strongly modulated by sensory feedback from receptors in the face and mouth, including trigeminal afferents from oral mechanoreceptors [13]. Importantly, oral sensory input may also contribute to the rhythmic regulation of toothbrushing itself. Uenoyama and Inada [34] reported that, during rolling-method brushing, blocking oral sensory perception disrupted the rhythmic pattern of brushing movement and muscle activity. Taken together, these observations make it plausible that chewing and brushing may be constrained, at least in part, by partially shared orofacial sensorimotor mechanisms, even though this does not imply that both behaviours are generated by the same central pattern generator.

Accordingly, the present findings should not be overstated as evidence for a shared rhythm-generating neural mechanism between chewing and toothbrushing. Unlike the simultaneous chewing–walking paradigm studied by Samulski et al. [33], chewing and brushing did not occur concurrently in our experiment. The observed association between chewing frequency and brushing stroke rate is therefore better interpreted as being consistent with a relationship at the level of oral motor tempo or broader individual timing tendencies, rather than as evidence for persistent coupling between distinct motor pattern generators. In contrast, the path through the dentition appears to be shaped primarily by learned and habitualized execution routines.

Several limitations should be considered. First, the chewing manipulation was acute and brief, so the present data do not address whether repeated exposure or longer-term sensorimotor training could alter brushing execution. Second, the sample consisted of younger healthy adults, limiting generalizability to other age groups and oral-health contexts. A logical next step would therefore be to relate cluster type and sequence organization to plaque levels or plaque removal. Finally, video recording may also have influenced behaviour to some extent. This possibility is supported by recent meta-analytic evidence showing that awareness of being recorded can alter participant behaviour, even when video-based observation is less intrusive than direct in-person observation [35]. A further limitation is that no independent manipulation checks of attention, alertness, arousal, stress, or relaxation were included. Although chewing and landscape-video viewing were selected as literature-informed experimental conditions, their immediate effects on participants’ internal state were not verified in the present study. Thus, it remains unclear whether the conditions induced short-term changes that did not translate into brushing behaviour, or whether they did not induce meaningful internal-state changes in the first place. Such measures were not included because the study aimed to observe habitual toothbrushing execution under standardized but minimally intrusive conditions, and additional questionnaires, attention tasks, or physiological recordings between the two brushing episodes could themselves have increased reactivity and influenced subsequent brushing behaviour.

A strength of the present study lies in the repeated within-subject assessment of toothbrushing execution under standardized recording conditions, allowing subject-specific stability to be examined across two closely matched time points. In addition to conventional surface-specific and temporal measures, the use of dynamic time warping enabled a more fine-grained comparison of brushing sequences and their structural similarity. This multimethod approach made it possible to capture not only how long subjects brushed or which surfaces they covered, but also how brushing execution was organized over time.

## Conclusion

The results of the study show that core features of toothbrushing execution, particularly surface-specific time allocation and spatiotemporal switching organization, were not meaningfully altered by prior chewing or landscape-video viewing. Across multiple observation points, toothbrushing behaviour showed high within-subject reproducibility, indicating strongly ingrained execution routines. At the same time, DTW-based sequence analysis and phenotype clustering demonstrated that these routines are not uniform across individuals, but are organized into distinct spatiotemporal brushing phenotypes. A further notable finding was the selective association between chewing frequency and brushing stroke rate. This linkage suggests a relationship at the level of oral motor tempo, but does not in itself demonstrate a shared rhythm-generating neural mechanism. Together, these findings support the view that toothbrushing should be understood not only as a frequently initiated health behaviour, but also as a structured form of habitual execution. This may help explain why toothbrushing can remain suboptimal despite being performed regularly and suggests that future oral hygiene interventions may need to target how brushing is executed over time and space, rather than focusing on duration alone.

## Supplementary Information

Below is the link to the electronic supplementary material.

Supplementary file 1

## Data Availability

All data supporting the findings of this study are available within the paper and its Supplementary Information.
