# Supplementary material for: Subject-specific stability of toothbrushing performance: insights from dynamic time warping and phenotype analysis
Source: Clin Oral Investig. 2026 Jul 16;30(8):335. doi: 10.1007/s00784-026-06997-3 (PMC13375744; doi:10.1007/s00784-026-06997-3)
Supplement: Supplementary file 1 [file 784_2026_6997_MOESM1_ESM.docx]

# Supplement Material

# Soft-bin representation and DTW

## 1) Starting point: interval coding in INTERACT

Toothbrushing behaviour was coded as time-stamped intervals (onset/offset in ms). Sextant and surface were annotated in separate event streams (Fig. S1A and S2A). At any time during the video, there is a current sextant label (1-6; for closed jaw brushing: 7 = 1_6, 8 = 2_5 and 9 = 3_4) and a current surface label (1 = oral, 2 = vestibular, 3 = occlusal), but they are stored in two separate timelines.

## 2) Creating one combined brushing-location timeline (time intersection)

To obtain a single, unified representation of brushing location, we intersected the sextant and surface timelines in time. Time was split wherever either the sextant label or the surface label changed, yielding contiguous segments labelled by the combination (sextant, surface). Because sextant has 9 possible codes and surface has 3 possible codes, there are 9 × 3 = 27 possible sextant×surface states.

## 3) Time normalization

Sessions can differ in total brushing duration. To enable comparison, the session timeline was rescaled to relative time (0%–100%) between the first intersected segment onset and the last intersected segment offset.

## 4) Dividing each session into 100 normalized time bins

Each session was divided into 100 equal-length bins along the normalized time axis. Thus, each bin represents 1% of that session, regardless of the absolute session duration.

## 5) Soft-bin representation: proportions of bin time

Within a bin, the brushing state may be stable or may change multiple times. Therefore, instead of forcing each bin to have a single label, we computed a “soft state” vector that retains mixtures. For each of the 27 states, we calculated the temporal overlap between the bin interval and all intersected segments labelled with that state, summed these overlaps, and divided by the bin length. This yields a 27-dimensional vector p = (p₁, p₂, …, p₂₇), where p_k is the proportion of bin time spent in state k and the values sum to approximately 1. (Bins with incomplete coverage due to gaps in the underlying coding may sum to <1; in our data coverage was typically near-complete.)

## Example (soft bin)

If bin 25/100 contains approximately 50% of its time in sextant 6, occlusal (S6×3) and 50% in sextant 6, oral (S6×1), then p_(S6×3) ≈ 0.5 and p_(S6×1) ≈ 0.5, with all other elements near 0. Importantly, the same 50/50 split in a different sextant (e.g., S5×3 and S5×1) produces a different vector because the mass lies in different coordinates.

## 6) The 100-bin sequence used for DTW

After repeating the above for all bins, each session is represented as a 100 × 27 matrix: rows correspond to bins (1..100) and columns correspond to the 27 sextant×surface states. Each row is a probability-like distribution over states for that bin (Fig. S1B and S2B).

## 7) Local bin-to-bin distance (cosine distance)

To compare two bins, we used cosine distance between their 27-dimensional vectors. Bins are most similar when they allocate time to the same sextant×surface states (cosine distance near 0) and most dissimilar when their time is spent in different locations (cosine distance toward 1).

## 8) Dynamic time warping (DTW)

DTW aligns two 100-bin sequences while allowing modest stretching/compression in time (to account for different pacing). DTW finds an alignment path through the 100×100 bin grid that minimizes the sum of local bin-to-bin costs, and we normalize the final DTW distance by alignment path length. Lower DTW values indicate greater similarity of spatiotemporal brushing patterns.

## 9) Dominant-state per bin (visualization only)

A 100×27 representation is difficult to visualize directly. For illustration, we also created a simplified “dominant state” timeline by selecting, in each bin, the state with the largest proportion (argmax_k p_k). This yields a 100-step discrete timeline (Fig. S1B and S2B). This dominant-state timeline is used only for visualization; DTW always uses the full soft vectors for all bins.

## 10) Interpretation of the DTW cost matrix and warping path (Fig. S1C and S2C)

For two sessions, DTW first computes a local cost matrix in which each cell contains the dissimilarity between one normalized time bin from session A and one normalized time bin from session B (here: cosine distance between the 27-dimensional soft state vectors). Colour encodes local cosine distance (lower = more similar), as indicated by the colour bar. DTW then identifies the minimum-cost warping path through this matrix from the first to the last bin, allowing horizontal, vertical, and diagonal steps that effectively “stretch” or “compress” time so that similar portions of the sequences are aligned. To prevent excessive warping, we constrained the alignment to a Sakoe–Chiba band (±20 bins for 100-bin sequences). A path close to the main diagonal indicates similar temporal progression between sessions, whereas deviations from the diagonal indicate local time shifts (e.g., one session spending relatively longer in a phase of brushing). The reported DTW distance corresponds to the average cost along the optimal path (path-length normalized), with lower values indicating higher similarity between sessions.


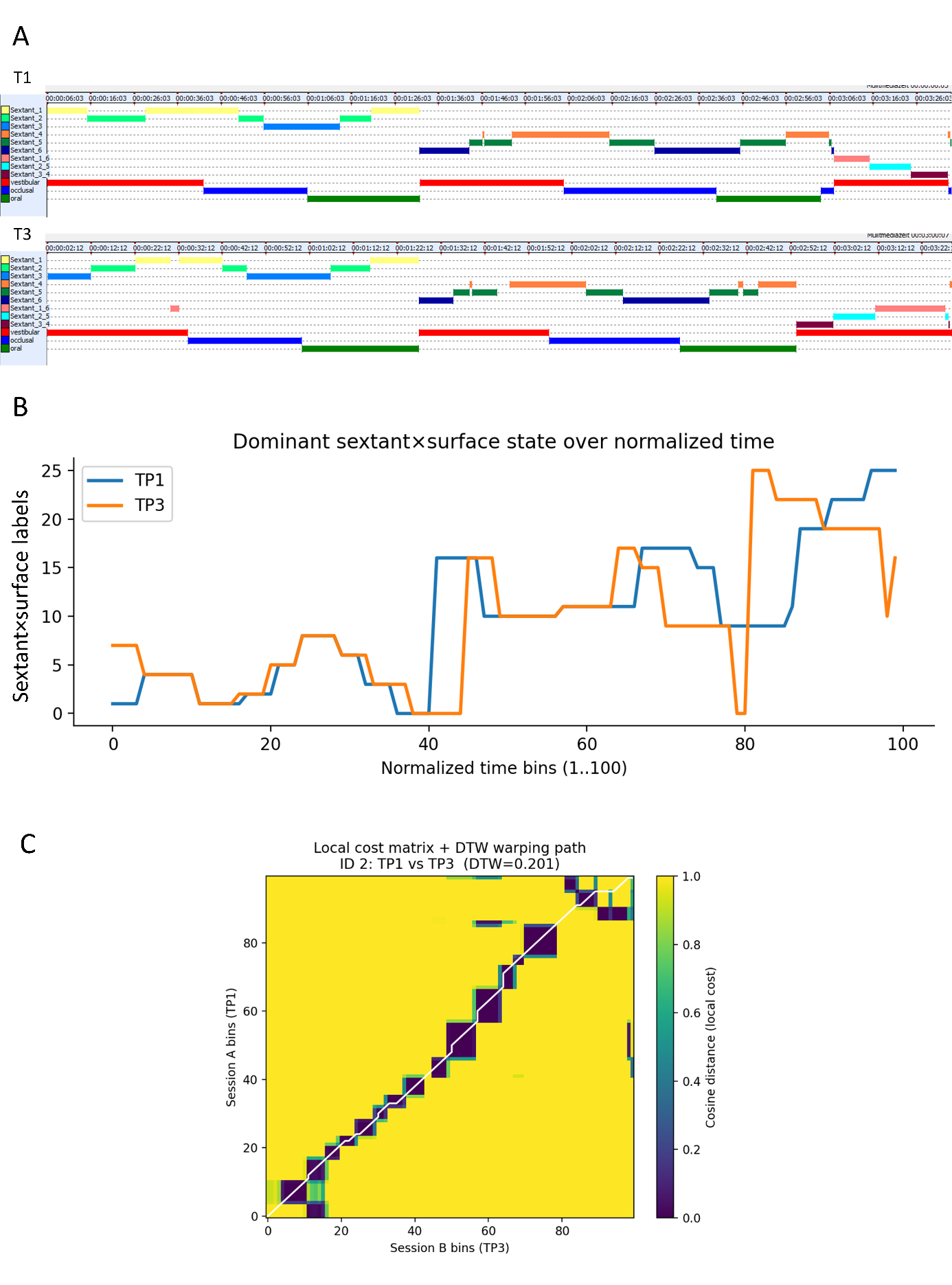


Fig. S1 Example of two very similar brushing events.

(A) INTERACT timelines showing time-stamped coding of sextant and surface in separate event streams.

(B) Intersected sextant×surface timeline for the same sessions, obtained by time-intersecting sextant and surface intervals to yield contiguous segments labelled by one of 27 states (9 sextant codes × 3 surfaces). Time is rescaled to normalized session time.

(C) Local cost matrix (cosine distance between 27-state soft vectors across 100 bins) with the DTW warping path overlaid. The path shows the optimal alignment between the two sessions; deviations from the diagonal reflect local time stretching/compression. The DTW value (path length normalised)

of 0.201 indicates high similarity.


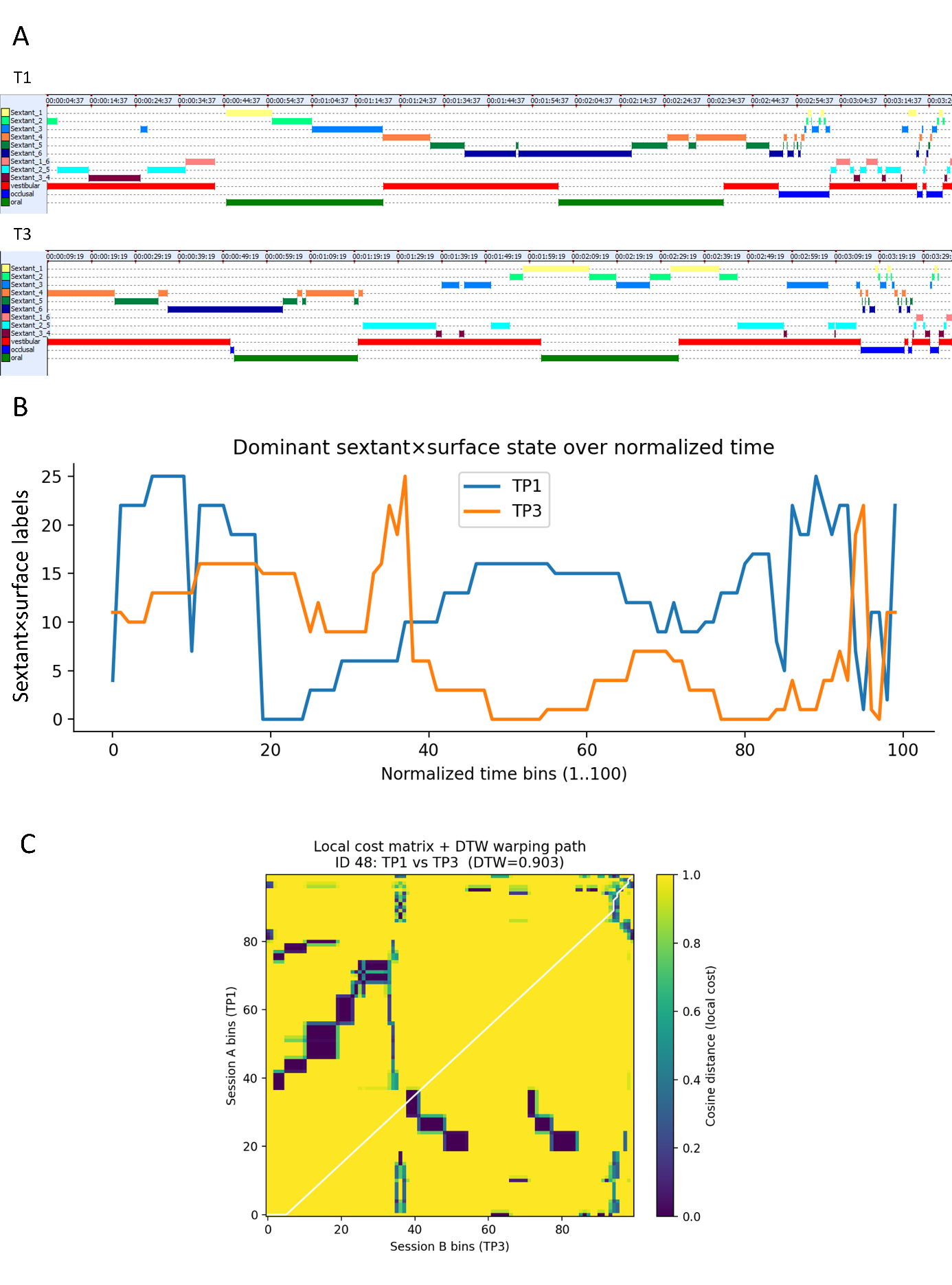


Fig. S2 Example of two dissimilar brushing events.

(A) INTERACT timelines showing time-stamped coding of sextant and surface in separate event streams.

(B) Intersected sextant×surface timeline for the same sessions, obtained by time-intersecting sextant and surface intervals to yield contiguous segments labelled by one of 27 states (9 sextant codes × 3 surfaces). Time is rescaled to normalized session time.

(C) Local cost matrix (cosine distance between 27-state soft vectors across 100 bins) with the DTW warping path overlaid. The path shows the optimal alignment between the two sessions; deviations from the diagonal reflect local time stretching/compression. The DTW value (path length normalised) of 0.903 indicates high dissimilarity.
